# Synthesis of water-soluble ionic terpolymers by inverse microemulsion and solution polymerization methods†

**DOI:** 10.1039/d2ra01173e

**Published:** 2022-04-25

**Authors:** Evelyn Y. Calvillo-Muñoz, Araceli Vega-Paz, Diego Guzman-Lucero, Irina V. Lijanova, Octavio Olivares-Xometl, Natalya V. Likhanova

**Affiliations:** Instituto Politécnico Nacional, CIITEC Cerrada Cecati S/N, Colonia Santa Catarina de Azcapotzalco CP 02250 CDMX México; Instituto Mexicano del Petróleo Eje Central Norte Lázaro Cárdenas No. 152, col. San Bartolo Atepehuacan, G. A. Madero 07730 CDMX México nvictoro@imp.mx; Benemérita Universidad Autónoma de Puebla, Facultad de Ingeniería Química Av. San Claudio y 18 Sur, Ciudad Universitaria. Col. San Manuel 72570 Puebla México

## Abstract

The synthesis of terpolymers can lead to very interesting combinations of monomers, which can affect the solubility of the polymer, its thermal stability or resistance in saline aqueous media. Free-radical inverse microemulsion and solution polymerization techniques were used to prepare water-soluble acrylamide-*N*-vinylpyrrolidone-(vinylbenzyl)trimethylammonium chloride terpolymers. The formulation of the polymerizable microemulsion was optimized by using the screening of surfactant percentage and HLB concept. The influence of synthesis temperature on the terpolymer composition and molecular weight was investigated. The reactions were carried out at 60, 70, and 75 °C for the microemulsion technique and at 40, 50, and 55 °C for the solution polymerization technique. The reaction products from both processes were water-soluble polymers, and the two techniques reached high conversions and molecular masses. Maximal molecular weights were displayed by terpolymers prepared by the solution method at 40 °C (959, 840 g mol^−1^) and the inverse microemulsion method at 60 °C (795, 994 g mol^−1^). According to NMR analysis, the highest amount of (vinylbenzyl) trimethylammonium chloride was incorporated into the terpolymer structure by the inverse microemulsion method. In contrast, the solution method yielded higher contents of acrylamide and *N*-vinylpyrrolidone. The viscosity properties of the terpolymers in aqueous solutions were directly correlated to their molecular weight and synthesis conditions.

## Abstract 2

Terpolymers based on acrylamide, *N*-vinylpyrrolidone and (vinylbenzyl)trimethylammonium chloride (VBTA) were synthesized using inverse microemulsion and solution methods. The microemulsion promoted the incorporation of VBTA in the terpolymer structure.

## Graphical abstract

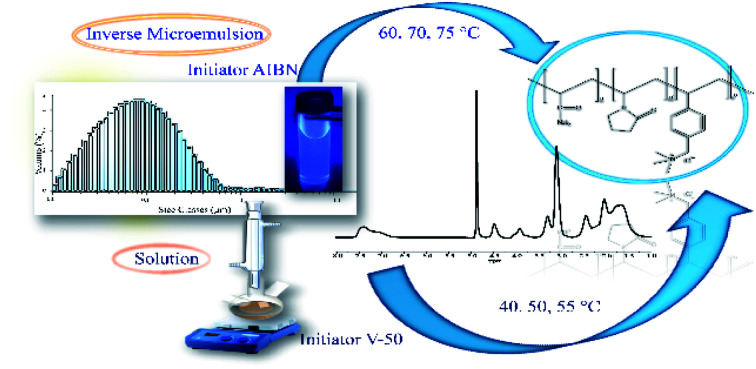

## Introduction

Currently, there is growing interest, especially in the oil industry, in the synthesis of hydrosoluble acrylamide-derivative co- or terpolymers with high molecular weights which are stable at high temperatures in the presence of saline media.^1–6^

The synthesis of these macromolecules can be achieved by different synthesis methods such as aqueous solution polymerization,^7–10^ mass polymerization, and inverse microemulsion polymerization.^11,12^ Nonetheless, each method features a series of advantages and disadvantages that have to be considered. One of the main characteristics of polymerization reactions is that they are highly exothermic, and a large amount of heat must be removed from the reaction medium. Another important aspect is the increase in the viscosity of the reaction system as conversion increases, which causes mixing, heat removal, and transport problems in the reaction mixture. Furthermore, polymerization reactions can be carried out using techniques whose polymerization medium is either homogeneous or heterogeneous. The advantage displayed by heterogeneous reaction polymerization systems, such as microemulsion, precipitation, or suspension, is that they are less sensitive to viscosity increase than the corresponding homogeneous systems under identical conditions, thus facilitating mixing, heat removal, and mass transport. So, the implementation of the polymerization technique depends on the engineering aspects and polymer properties.^13^ However, inverse microemulsion polymerization is an effective method to produce controlled polymers with high molecular weight, which is a characteristic that helps overcome the problems associated with the high viscosity levels reached in bulk or solution methods.^14^

The microemulsion method allows not only to obtain polyacrylamides or polyacrylates with high molecular weight up to 20 × 10^6^ Da (ref. 15–17) by RAFT polymerization^18^ or controlled/living radical polymerization,^19^ but also acrylamide copolymers^2,20–22^ and terpolymers^6,20,23–25^ even by the semicontinuous or continuous methods.^26–31^

So, copolymers based on acrylamide and quaternary ammonium dimethylaminoethyl methacrylate or dimethylaminoethyl acrylate have been synthesized by means of inverse emulsion polymerization in batch and semi-batch reactors using 3–4% of emulsifier and azobisisobutyronitrile (AIBN)^32^ and dual redox ammonium persulfate/sodium bisulfite as initiators.^33^

As for cationic water-soluble polymers, they have attracted scientific attention due to their resistance in saline environments. In this sense, polyelectrolytes based on *N*-isopropylacrilamide, 2-acrylamido-2-methylpropanesulfonate, and 2-acrylamido-2-propanetrimethylammonium chloride were obtained with molecular weight of approximately 1 million Da from polymerizable microemulsions with 12–18% of emulsifier concentration, HLB = 9.4, and 0.03 mol% of redox initiator.^34^ Although there are several articles on the synthesis of copolymers based on (vinylbenzyl)trimethylammonium chloride (VBTA), most of them are about latex preparation.^35–38^ Unfortunately, the works describing the water-soluble synthesis of co- or terpolymers based on VBTA are very scarce.^39,40^ The incorporation of the ionic unit (in this case VBTA) to the structure of the water-soluble terpolymer promotes stability in a salt medium, while the incorporation of *N*-vinylpyrrolidone favors the thermal stability of the polymer, and the presence of acrylamide provides solubility in water and viscosity to the final polymer solutions, for which the search for synthesis processes of ionic terpolymers with molecular weights greater than 0.5 × 10^6^ Da is very current.

**Fig. 1:**
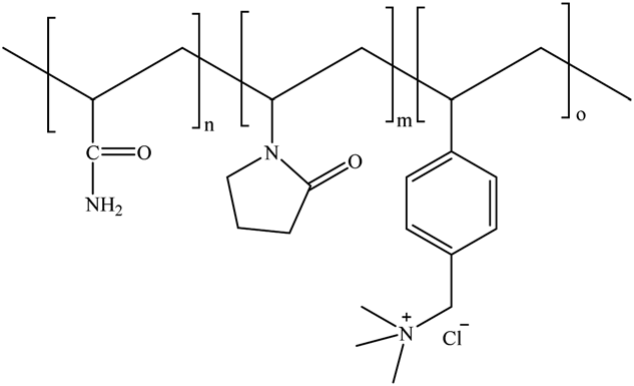
Structure of the synthesized AM/VP/VBTA (50/30/20) terpolymer.

Therefore, in this work, successful examples of the synthesis of water-soluble acrylamide-*N*-vinylpyrrolidone-(vinylbenzyl)trimethylammonium chloride terpolymers using free-radical inverse microemulsion and solution polymerization techniques were reported and the comparison between both polymerization methods was made. The effects of the polymerization parameters (temperature and type of initiator) on the molecular weight, composition and thermal stability properties of the polymer were studied (Fig. 1). The inverse microemulsions were prepared as a function of the hydrophilic–lipophilic balance (HLB), selecting the most favorable polymerization conditions.

## Experimental part

### Materials

All reagents were analytical grade and purchased from Sigma-Aldrich. Acrylamide, (AM, ≥98%), *N*-vinylpyrrolidone (VP, ≥99%), VBTA (99%), 2,2′-azobis(2-methylpropionamidine)dihydrochloride (V-50, 97%) and AIBN (12 wt% in acetone) were used as initiators; *n*-decane (≥98%) was used as the continuous phase of the inverse emulsion, and Igepal CO 720 and Span 80 were used as emulsifiers.

### Terpolymer synthesis by the solution method

Polymerizations were carried out in a three-neck round-bottomed flask, fitted with a mechanical stirrer and water condenser, immersed in an oil bath at the corresponding temperature. The reaction was carried out at a constant pH value of 9 by adding 1 M NaOH to avoid VP hydrolysis. Monomers were added at the desired molar ratio of 50/30/20 (AM/VP/VBTA), respectively, to 50 mL of deionized water previously degassed for 30 min in vacuum and the total concentration of solids was 1.5 mol L^−1^. The reaction was carried out under nitrogen flow at 45, 50, and 55 °C at 150 rpm for 30 min before introducing the initiator V-50 (0.5 mol% in 10 mL of water; 0.007 initiator mole ratio of monomers) at 30 mL h^−1^ with a syringe pump. The reaction was allowed to proceed for 8 h. After finishing the reaction, the solution was precipitated in acetone, re-dissolved in water, and re-precipitated in acetone with final polymer yields between 60 and 70%. The terpolymer was dried in a vacuum oven at 50 °C for 24 h.

### Terpolymer synthesis by the inverse microemulsion method

The continuous lipophilic phase of the inverse emulsion was prepared by dissolving 1.0 g of Igepal CO 720 in 16 mL of *n*-decane. In sequence, 3.3 g of Span 80 with 1.27 g of AM, 1.20 g of VP, and 1.53 g of VBTA monomers (50/30/20 ratio of AM/VP/VBTA) were dissolved in 6 mL of degassed water. Both phases were mixed using an Ultra-turrax T25 Basic homogenizer at 13 000 rpm for 8 min. The size of the particles was measured by using an AccuSizer 780 AD Autodiluter. Then, 26 mL of microemulsion were poured into a three-neck round-bottomed flask and deaerated by bubbling nitrogen for 30 min. Under nitrogen atmosphere and constant stirring at 350 rpm, 54 μm of the initiator AIBN (0.007 initiator mole ratio of monomers) was added at 30 mL h^−1^ using a syringe pump. The polymer synthesis was carried out at 60, 70, and 75 °C. Nitrogen flow was kept for 8 h of the polymerization reaction. After finishing the reaction, acetone was added to the reaction mixture. The formed product was re-dissolved in water and re-precipitated in acetone with final polymer yields between 60 and 70%. The terpolymer was dried in a vacuum oven at 50 °C for 24 h.

### Characterizations

^1^H NMR spectroscopy analyses were performed on a Bruker 600 MHz spectrometer with Avance III as interface. Intrinsic viscosities were evaluated in an Ubbelohde glass capillary viscometer at 25 °C using 0.5, 0.3, and 0.2 wt% of terpolymer concentration in a 0.1 M NaCl aqueous solution. The terpolymers' glass transition (*T*_g_) was determined using the cyclic DSC technique in the Setaram DSC131 EVO equipment, under a heating ramp of 5 °C per min in N_2_ atmosphere. As part of the first heating cycle for removing waste or solvents, the temperature was increased from 25 to 200 °C and then decreased to 25 °C. The heating and cooling conditions were performed under the above conditions for the second cycle.

Then, the *T*_g_ of all the terpolymers was obtained for the second heating cycle. Conventional DSCs were carried out by means of a Netzsch piece of equipment under nitrogen atmosphere using a heating ramp of 5 °C min^−1^. The thermal properties were determined by high-resolution thermogravimetric analysis (TGA) using a Netzsch STA 409 in a nitrogen environment at a heating rate of 5 °C min^−1^. Molecular weights were measured in a GPC Agilent series 1260 Infinity equipment with 2 Aquagel-OH Mixed-H 8 μm 300 × 7.5 mm columns connected in series. A Hamielec calibration was made for three broad polyacrylamide standards: low molecular weight *M*_w_ 79 900 Da and PD of 1.8, medium molecular weight *M*_w_ 1 140 000 Da and PD of 2.45, and high molecular weight *M*_w_ 5 550 000 and PD of 10.37. Fourier transform infrared analyses were measured in a Thermo Scientific Nicolet 8700. The molar composition of the repeating units of the terpolymers was calculated using ^1^H NMR. For the VBTA unit, the peak at 4.49 ppm was used (*A*_*i*_^*δ*^), which is integrated for 2H (No. *H*_*i*_^*δ*^), and for VP, the one at 3.91 ppm is also integrated for 2 protons (Fig. 5). For these two units, eqn (1) was used to obtain the equivalent area of the respective protons (*H*^E^_*i*_):1
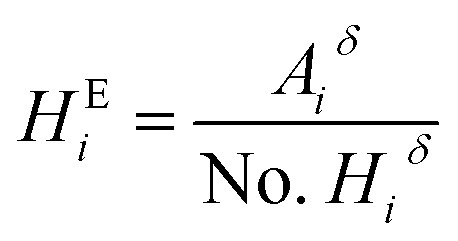


For the AM unit, the equivalent area was calculated using eqn (2), integrating the area between 0.94–2.78 ppm, where 3H of AM, 6H of VP, and 3H of VBTA were found:2
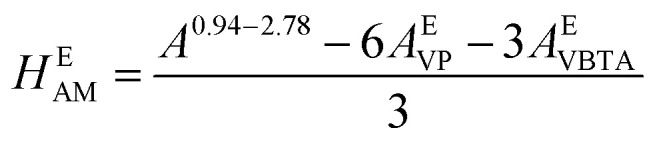


The molar percentage of the repeating units (%mol_*i*_) was obtained using eqn (3), where each equivalent area of the protons (*H*^E^_*i*_) was divided by the sum of the three equivalent areas (*H*_T_):3
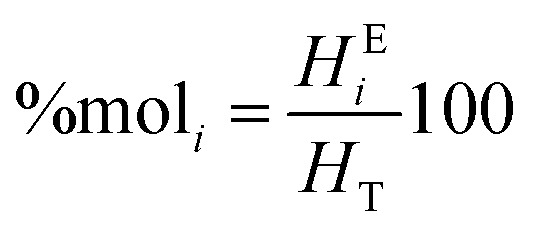


## Discussion

### Microemulsion preparation

Microemulsions appear as homogeneous, transparent solutions. The oily and aqueous phases are fundamental to form the microemulsion since water solubilizes a polar core of surfactants, forming the so-called water pool.^41,42^ In contrast, the oil phase with an appropriately long surfactant chain easily embeds in the interface between the oil and water layers to form a stable micellar microemulsion.^43^ Nevertheless, the HLB property of any surfactant is a determining condition for the formation of W/O type microemulsions. According to classical theory, for the construction of stable W/O systems, HLB values from 3 to 6 are required, while O/W emulsions can be formed with surfactants with HLB numbers between 8 and 18.^44^ The problem with HLB is that its predictive power is low,^44,45^ and it is possible to prepare W/O emulsions at HLB values around 7.^43,46^ Therefore, the most stable emulsion systems usually consist of mixtures of two or more emulsifiers, one part with lipophilic tendencies and the other with hydrophilic features. By blending both emulsifier types, the exact needed HLB can be achieved.

**Fig. 2:**
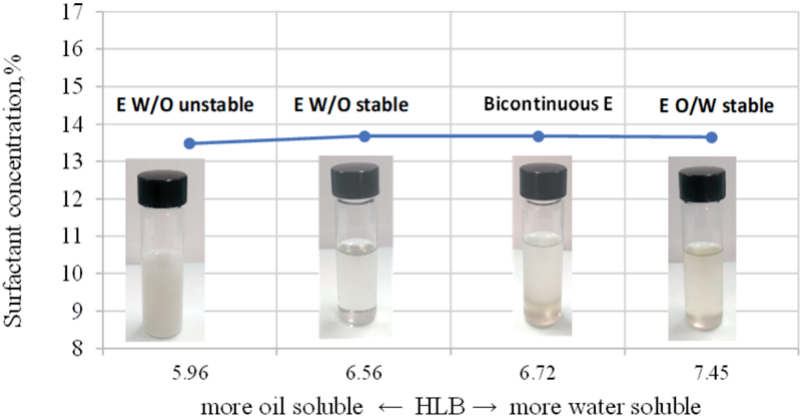
Relationship between HBL and type of formed microemulsion.

There is a synergistic effect on solubilization by mixing a highly oil-soluble surfactant and a highly water-soluble surfactant. The mixture of two surfactants makes it possible for these components to be preferentially distributed at the interface, reaching a high solubilization level.^47^ In the present study, two nonionic and polymeric surfactants, Span80 and Igepal CO 720, were used. These surfactants stabilize the W/O microemulsions through the steric effect when the surfactant is adsorbed on the dispersed water droplets covering them. In addition, the surfactant tail prevents the droplets from coming into contact with each other.^48,49^Fig. 2 shows the formed microemulsion type as HLB function of the surfactant mixture, composed of Span 80 and Igepal CO 720, where at values below 6.7 of HLB, microemulsions water-in-oil type is formed. In contrast, at HLB values greater than 7, there is a tendency to form microemulsions of the oil-in-water type.

**Fig. 3:**
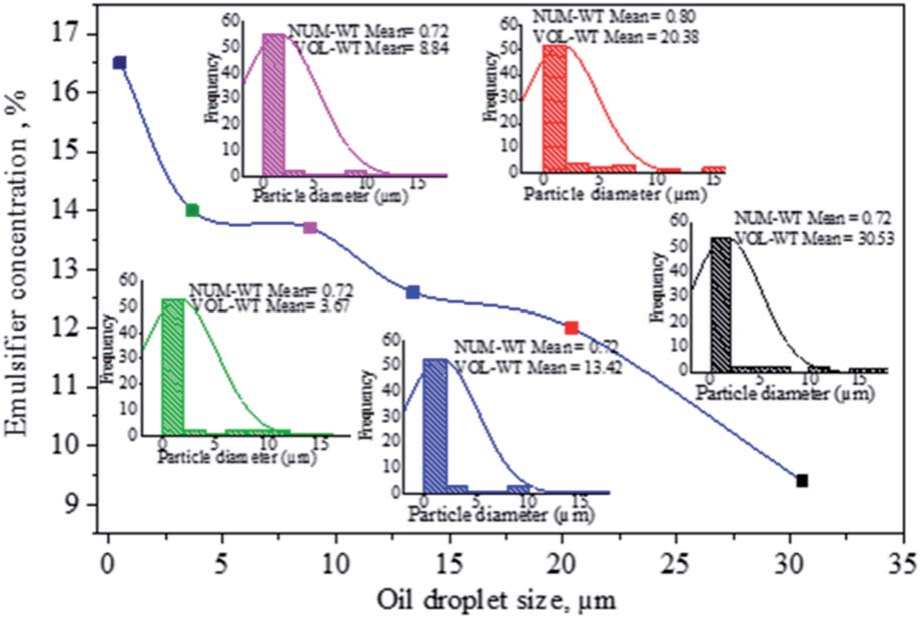
Oil droplet evolution as a function of emulsifier concentration.

The influence of surfactant concentration is based on displaying droplet populations and determining average droplet size values for emulsions. A decrease in average droplet diameter with increasing emulsifier concentration is attributed to decreasing interfacial tension between the dispersed aqueous droplets and surrounding oil medium, decreasing the size of the droplets below 3 μm (Fig. 3). Therefore, higher amounts of surfactant result in smaller drop sizes and more significant final conversion.^50^

**Fig. 4:**
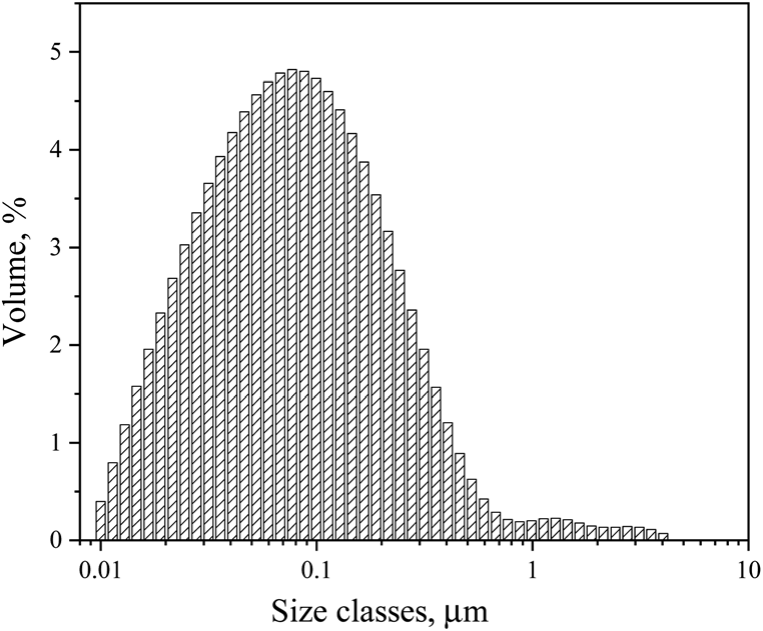
Diameter size of dispersed phase of the microemulsion at 16.5% of surfactant concentration and HBL of 6.56.

Finally, with 16.5% of surfactant concentration at HLB of 6.56, it was possible to form a transparent microemulsion with a drop size below 0.1 μm (Fig. 4). This result is consistent with the research work by Mahdi, who found that HLB of 6.31 for the two-emulsifier system, Span 80 and Tween 85, was optimal for the synthesis of polyacrylamide by the inverse microemulsion polymerization method.^51^

### Polymerization process and characterization

The microemulsion and solution polymerizations of terpolymer by keeping constant the 50/30/20 molar ratio of the AM/VP/VBTA monomers and other reaction parameters (initiator, reaction time) were carried out. The main parameters that were changed were the type of initiator and temperatures (Table 1).

**Table 1:** Terpolymerization reaction conditions

Variable	Microemulsion	Solution
Reaction temperature, °C	60, 70, 75	40, 50, 55
Concentration of total solids, mol L^−1^	1.5	1.5
Initiator concentration, mol initiator/mol monomers	0.007	0.007
Initiator type	AIBN	V-50
Molar ratio of reagents (AA–VP–VBTA)	50–30–20	50–30–20
Amount of total water, mL	6	50
Amount of total *n*-decane, mL	16	0
Reaction time, h	8	8
Dosing speed	30 mL h^−1^	30 mL h^−1^
pH	6	9

In the case of polymerization in an aqueous solution, the water-soluble initiator V-50 was used, while the oil-soluble initiator AIBN was employed for the inverse microemulsion polymerization. Baade and Reichert suggested a dispersion polymerization model for the acrylamide system. Their model revealed that radicals from the oil phase diffused into the water phase in which polymerization took place when the oil-soluble initiator was used.^52^

**Fig. 5:**
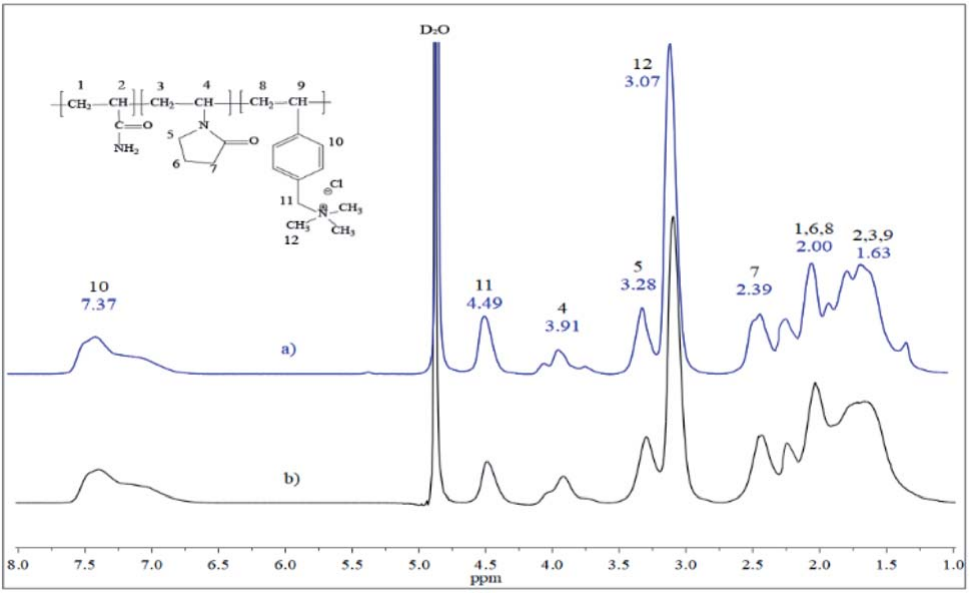
^1^H NMR terpolymers: (a) microemulsion at 70 °C and (b) solution at 50 °C.

The transparent inverse microemulsion with a concentration of the surfactant mixture Span 80 and Igepal CO 720 equal to 16.5% at HLB of 6.56, *n*-decane as the continuous phase and water with dissolved monomers as the dispersed phase, with drop size below 1 μm, was used for polymer synthesis. For both polymerization reactions, 1.5 mol L^−1^ of the total concentration of solids and 0.007 initiator mole ratio of total monomers were used. The pH was always adjusted to 9 using the NaOH solution by the solution synthesis method. In contrast, by the inverse microemulsion method, the pH was kept equal to 6 without further pH adjustment. The reaction was carried out at three different temperatures (60, 70, 75 °C) for inverse microemulsion and 45, 50, and 55 °C for solution polymerization. Three temperatures and different initiators for each method were chosen, so AIBN is recommended to use in the temperature interval between 60 to 80 °C. In contrast, V-50 is used between 40 and 60 °C.^17,18,35,37^ The terpolymer molar compositions were calculated through the ^1^H NMR analysis (Fig. 5 and Table 2).

**Table 2:** Terpolymer molar composition by microemulsion and solution polymerization methods

Microemulsion				Solution			
Temperature, °C	Terpolymer unit, mol%			Temperature, °C	Terpolymer unit, mol%		
	AA	VP	VBTA		AA	VP	VBTA
60	56	25	19	40	61	29	10
70	53	27	20	50	59	28	13
75	58	24	18	55	58	30	12

As shown in Table 1, solution polymerization produced terpolymers with the highest VP incorporation (28–30 mol%), while the inverse microemulsion polymerization method resulted in polymers with the highest amount of ionic part (18–20 mol%). There was higher incorporation of acrylamide units than VP and VBTA units, primarily when the solution polymerization method was used, reaching 58–61 mol% concentrations. As for the microemulsion polymerization method at 70 °C, the concentration of monomeric units in the final polymer (53/27/20) was very similar to the initial concentration in the starting reaction mixture (50/30/20). The ^1^H NMR spectra of the terpolymers synthesized by both methods are similar (Fig. 5). The signals at the 1.20–2.55 ppm region belong to the main polymer chain formed by acrylamide, AM (1 and 2), VBTA (8 and 9) and VP (3), where the VP lactam ring signals correspond to (6 and 7). The signals of the VBTA aromatic ring are located at 7.37 ppm. The main peak (12) at 3.07 ppm belongs to methyl groups linked with VBTA quaternary ammonium, where the signal (11) at 4.49 ppm is attributed to the VBTA methylene group. The broad signals at 3.91 (4) and 3.28 ppm (5) correspond to the terpolymer VP unit. The above analysis shows that the expected chemical structures were obtained.

The acrylamide conversion was favored in both polymerization methods. The conversions were calculated based on NMR data using the vinyl proton integration of the monomers (Table 3). For the solution method, they were 96, 94, and 93% at 40, 50, and 55 °C, respectively. As for the microemulsion method, they were 90, 87, and 92% at 60, 70, and 75 °C, respectively. It can also be noted that the VBTA monomer had the lowest conversion when the solution method was used with 48, 62, and 57% at 40, 50, and 55 °C, respectively. In contrast, the VBTA conversion was significantly increased when the microemulsion method was used; it reached 95, 98, and 85% at 60, 70, and 75 °C, respectively. Finally, VP got higher conversion in solution method: 93, 89, and 96% (at 40, 50, and 55 °C) than with the microemulsion method, which obtained 78, 84, and 75% (at 60, 70, 75 °C).

**Table 3:** Monomer conversion by the ^1^H NMR method

Microemulsion			Solution		
Temperature, °C	Monomer	Monomer conversion, %	Temperature, °C	Monomer	Monomer conversion, %
60	AA	90	40	AA	96
	VP	78		VP	93
	VBTA	95		VBTA	48
70	AA	87	50	AA	94
	VP	84		VP	89
	VBTA	98		VBTA	62
75	AA	92	55	AA	93
	VP	75		VP	96
	VBTA	85		VBTA	57

The molecular weight of the terpolymers decreased as the reaction temperature increased (Table 4). It is known that, as the temperature increases, the concentration of radicals grows and, therefore, the reaction rate rises and the molecular weight decreases.^53^ The polymers that were synthesized by the solution method presented higher molecular weights (from 959.840 to 576 569 g mol^−1^) than those obtained by microemulsion (from 795 994 to 680 927 g mol^−1^) (Fig. S1a and b†). This behavior could be attributed to acrylamide mainly being incorporated in the solution method (Table 2), generating higher molecular weights than the other two monomers. The higher the molecular weight, the higher the intrinsic viscosity. It was 4.8 dL g^−1^ for the terpolymer synthesized by the solution method. In free radical polymerization, reactions with high molecular weight and conversion, the distributions become much broader than those that lead to low conversions and molecular weight.^13^ The present study obtained the highest polydispersity index for the polymers synthesized in the solution method and grew with increasing reaction temperature. They were 2.8 for the terpolymer synthesized at 55 °C.

**Table 4:** Molecular weight and viscosity of the terpolymers

Temperature, °C	Molecular weight, g mol^−1^	PD	*η*_Int_, mPa s
**Microemulsion**			
60	795 994	1.5	3.1
70	717 954	1.4	2.8
75	680 927	1.6	2.5

**Solution**			
40	959 840	1.9	4.8
50	693 907	2.0	2.7
55	576 569	2.8	2.4

### FTIR and thermal analysis

**Fig. 6:**
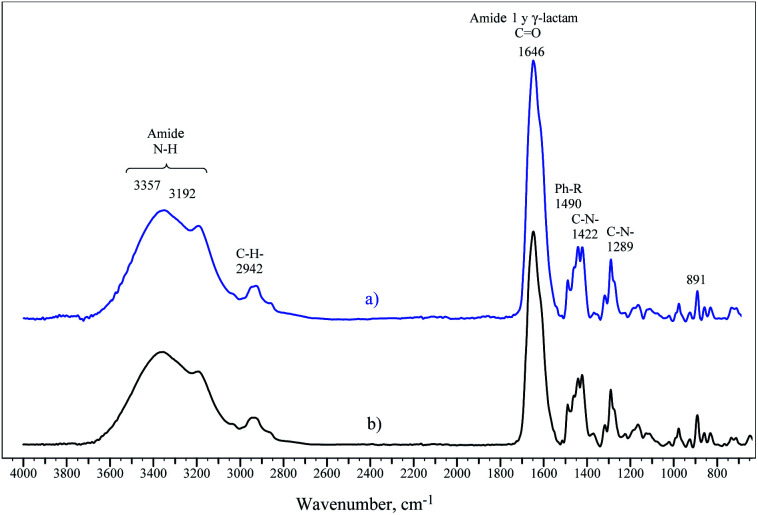
FTIR spectra of the synthesized terpolymers: (a) microemulsion at 70 °C and (b) solution at 50 °C.

Fig. 6 illustrates the FTIR spectra of terpolymers synthesized by the microemulsion and solution methods, which are very similar. Signals of *ν*(NH) appear between 3192–3357 cm^−1^. The signal at 2942 cm^−1^ corresponds to the *ν*(CH_2_) group. The characteristic (C–C) peaks from the aromatic ring appear at 1490 cm^−1^, while the band at 1422 cm^−1^ corresponds to *ν*(C–N) bond vibrations, which may belong to the amide quaternary ammonium groups. The FTIR spectra region at 1355–1394 cm^−1^ corresponds to the *ν*(CN) signal of the VP lactam group, and the signal at 891 cm^−1^ belongs to the quaternary ammonium in VBTA.

**Fig. 7:**
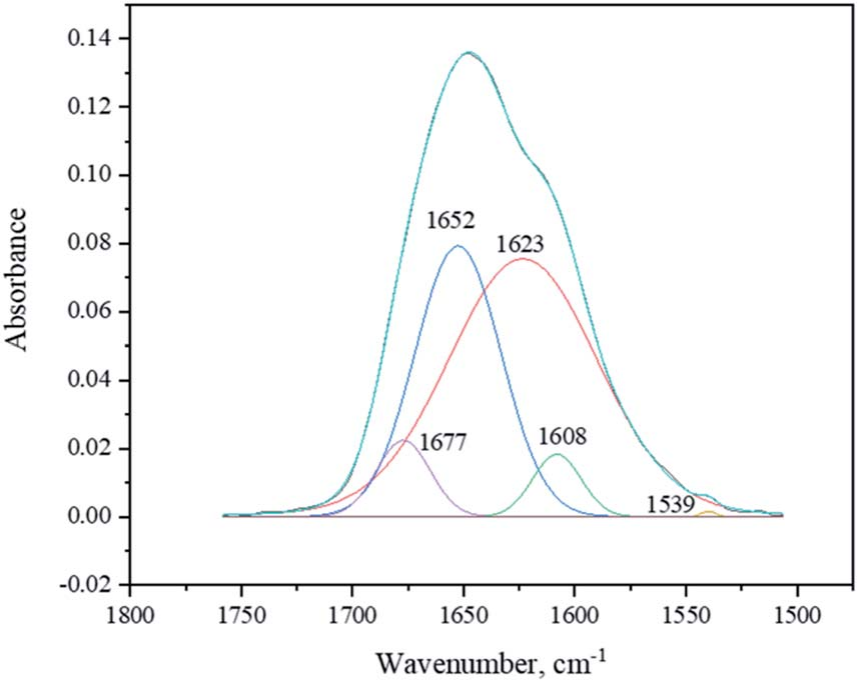
FTIR deconvoluted signal at 1500–1800 cm^−1^.

The deconvolution of the signal at 1500–1800 cm^−1^ with the maximum at 1646 cm^−1^ produced the set of 5 sub-peaks: *δ*(CHN aromatic) at 1539 cm^−1^, *ν*(C–Carom) at 1608 cm^−1^, *δ*(NH amide) at 1623 cm^−1^, *ν*(C=O amide) at 1652 cm^−1^, and *ν*(C=O lactam) at 1677 cm^−1^ (Fig. 7).

**Fig. 8:**
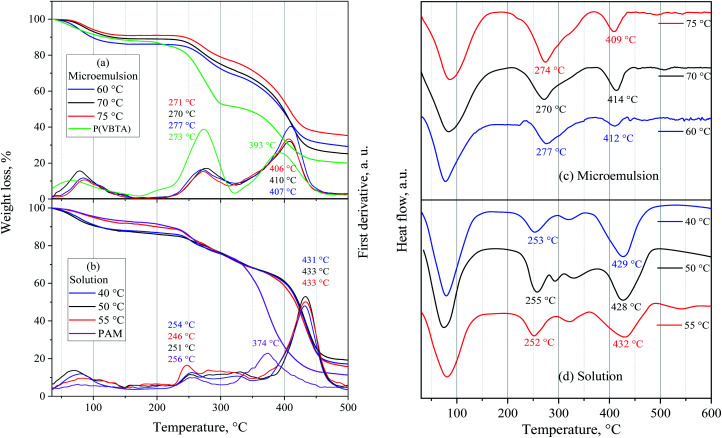
TGA and DSC of the synthesized terpolymers by (a) and (c) inverse microemulsion and, (b) and (d) solution polymerization.

The thermal analysis of the terpolymers revealed that all these polymers lost weight in nitrogen at three well-defined stages (Fig. 8 and Table 5). The first weight loss corresponds to the elimination of the solvent, in this case, water. The terpolymers lost between 9 and 14 wt% of their weight. The second stage of weight loss is associated with the thermal degradation of the terpolymers. It is known that acrylamide units decompose, releasing ammonia and forming imide groups.^54,55^ The stability of PAM has been reported up to 285 °C^54^ and 326 °C.^56^Fig. 8 shows that the starting decomposition temperature of PAM was 228 °C, and it is very close to that reported by Leung *et al.*, which was 220 °C.^57^ The difference between the high degradation temperatures reported and obtained in this study is that the reported samples were dried at 210 °C, and the ones featured in this work were dried at 50 °C for 24 h. The P(VBTA) homopolymer began the decomposition stage at 219 °C and was associated with weight loss of 33 wt%. The weight loss corresponds to the loss of trimethylamine groups.^58,59^ The terpolymers exhibited the second weight-loss stage in the range of homopolymers. For the terpolymers that were obtained by the microemulsion technique, the weight loss was between 14 and 18 wt%, and the temperature values for the first derivative peak were found between 270 and 277 °C. This decomposition was also observed in the DSC curves between 270 and 277 °C (Fig. 8c). For the terpolymers that were obtained by the solution technique, the weight loss was between 19 and 20 wt%, and the temperature values of the derivative peak were between 249 and 256 °C. In the DSC, these thermal events are between 252 and 255 °C (Fig. 8d). It can clearly be seen that the polymers obtained by solution have lower decomposition temperatures and are closer to PAM decomposition temperature. This can be attributed to the fact that the terpolymers obtained by the solution technique have a higher composition of acrylamide units, between 58 and 61 mol% (microemulsion from 53 to 58 mol%), and lower content of VBTA units, between 10 and 12 mol% (microemulsion from 18 to 20 mol%) (Table 1). The third weight loss stage corresponds to the final degradation of the terpolymers. The temperature at the derivative peak for the terpolymers obtained by microemulsion was between 406 and 410 °C, and for those obtained by solution, it was found at 433 °C.

**Table 5:** Weight loss temperature by TGA and *T*_g_ for the terpolymers^a^

Synthesis temperature, °C	*T*_g_, °C	Humidity, %	*T*_s_, °C	*T*_p1_, °C	Weight loss second stage, %	*T*_p2_, °C
**Microemulsion polymerization**						
60	140	14	250	277	17	407
70	136	11	244	270	18	410
75	142	10	244	271	14	406

**Solution polymerization**						
40	148	12	238	254	18	431
50	145	13	240	251	20	433
55	143	9	232	246	19	433

a *T*_s_: weight loss starting temperature, *T*_p1_: first temperature at first derivative peak, *T*_p2_: second temperature at first derivate peak.

This weight loss was related to DSC endothermic events between 409–414 °C for microemulsion and between 428 and 432 °C for those obtained by solution (Fig. 8c and d). This difference between the terpolymers synthesized by the two polymerization techniques could be because those obtained by the solution technique have higher VP units (28–30 mol%) in their macrostructure, compared to those that were obtained by solution (24–27 mol%). According to the previous results, it can be said that the higher VP content, the higher the temperature at the derivative peak of the third decomposition stage.

The *T*_g_ of the terpolymers was obtained from the DSC second heating cycle up to 200 °C to eliminate most of the water and avoid the plasticizing effect.^60^ As shown in Table 5, the terpolymers polymerized in solution have higher *T*_g_ than those that were polymerized in microemulsion. Polymers with higher *T*_g_ have higher polymer–polymer interactions, generating lower macro chain mobility and consequently an increase in *T*_g_. The amide groups are promoters of these interactions.^61,62^ For this reason, the terpolymers with higher acrylamide composition tend to have higher *T*_g_. In addition to the above, it can also be observed that the terpolymers obtained by the solution technique have lower VBTA content. This repeating unit with the benzyl trimethyl ammonium group separates the macro chains, and the *T*_g_ tends to decrease. Therefore, the VBTA concentration in the terpolymers, as seen in Table 5, also modifies the *T*_g_ value. Consequently, the highest *T*_g_ was for the terpolymers with higher concentrations of acrylamide units and lower concentrations of VBTA units. As a result, the terpolymers obtained by the solution technique had higher *T*_g_ (143–148 °C) than those values obtained by the microemulsion technique (136–142 °C).

**Fig. 9:**
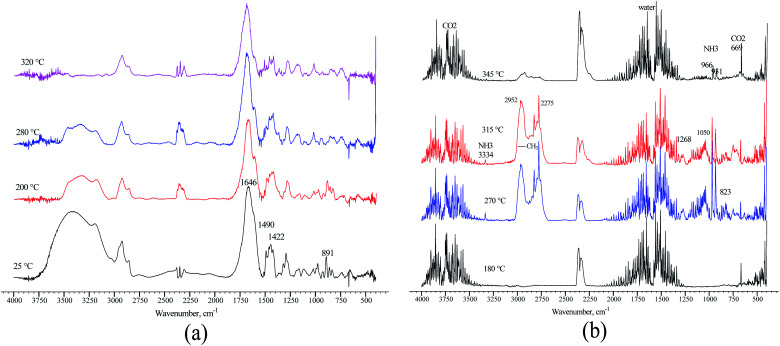
FTIR spectra of the terpolymer obtained by the inverse microemulsion method: (a) recorded at different temperatures; (b) the exhaust gases of terpolymer decomposition.

**Fig. 10:**
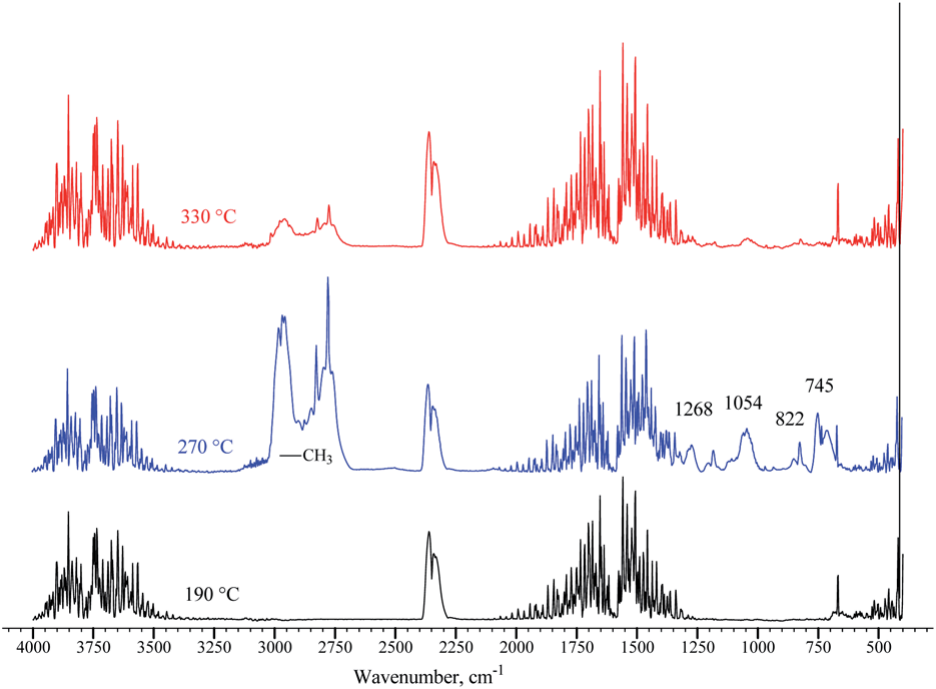
FTIR spectra of P(VBTA) decomposition gases.

The loss of amide and trimethylamine groups during the thermal decomposition process of the polymer molecule was observed through monitoring. A terpolymer sample obtained by microemulsion methods at 70 °C was placed in a reaction chamber and heated at four temperatures: 25, 200, 280, and 320 °C. The FTIR spectrum changes in the terpolymer sample are shown in Fig. 9a. When the temperature was increased up to 320 °C, the bands at 891, 1422, 1490, and 1646 cm^−1^ underwent more significant changes, which means that the amide and quaternary ammonium groups presented thermal decomposition. The exhaust gases of terpolymer decomposition are shown in Fig. 9b. It shows that trimethylamine and ammonium are the main evolving compounds when heating the sample up to 270 °C, associated with the second stage of TGA terpolymer weight loss. There are also carbon dioxide and water vapors whose concentration increased with increasing temperature; furthermore, the decarboxylation of the amide group occurred. Carbon dioxide and water are straightforward to be identified; CO_2_ showed strong absorption bands in the 2320–2400 cm^−1^ region, at 669 cm^−1^, and overtones at 3590–3740 cm^−1^, while water bands appeared in the 1300–1800 cm^−1^ region. Prominent ammonia gas bands are located at 931, 966, and 3334 cm^−1^, corresponding to amide group decomposition. Trimethylamine vapor appeared very strong at 1268 cm^−1^, 1180–1050 cm^−1^, and 823 cm^−1^ at 270 °C,^62^ disappearing as the temperature increased from 315 to 345 °C. Similar behavior was observed in the bands of the methyl group at 2952 and 2275 cm^−1^ that belong to VBTA. However, these absorptions almost wholly disappeared at 345 °C when the second stage of thermal degradation was completed. This degradation stage correlates quite well with the exhaust decomposition gas spectrum of the VBTA homopolymer (Fig. 10).

## Conclusions

The synthesis of water-soluble terpolymers, with molecular weight around 0.5–1 × 10^6^ g mol^−1^, featuring AM, VP and VBTA units in their structure, which provided them with properties such as thermal stability, water solubility and viscosity, was achieved by means of two free-radical methods: inverse microemulsion and solution. The inverse microemulsion was prepared with a mixture of Igepal CO 720 and Span 80 surfactants at a total concentration of 16.5% at HLB of 6.56, monomers, water, and *n*-decane with drop size below 0.1 μm. The inverse microemulsion method did not need pH adjustment during the reaction process, while the solution method required pH adjustment at 9 during the synthesis reaction. Characterization tests such as NMR, FTIR and TGA confirmed the presence of AM, VP and VBTA in the terpolymer chemical structure. The terpolymers synthesized by the solution method accommodated a greater amount of VP and AM in their structure. In contrast, the inverse microemulsion method promoted the incorporation of VBTA in the terpolymer structure.

## Author contributions

Evelyn Y. Calvillo-Muñoz: conceptualization, methodology, validation, investigation, writing – original draft. Araceli Vega-Paz: methodology, validation, formal analysis. Diego Guzman-Lucero: formal analysis, writing – original draft. Octavio Olivares-Xometl: formal analysis, investigation. Irina V. Lijanova: conceptualization, investigation, supervision. Natalya V. Likhanova: conceptualization, investigation, writing – review & editing, project administration.

## Conflicts of interest

There are no conflicts to declare.

## Supplementary Material

RA-012-D2RA01173E-s001
