# Supplementary material for: Synthesis of water-soluble ionic terpolymers by inverse microemulsion and solution polymerization methods
Source: RSC Adv. 2022 Apr 25;12(20):12273–82. doi: 10.1039/d2ra01173e (PMC9036531; doi:10.1039/d2ra01173e)
Supplement: RA-012-D2RA01173E-s001 [file RA-012-D2RA01173E-s001.pdf]

Electronic Supplementary Material (ESI) for RSC Advances.
This journal is © The Royal Society of Chemistry 2022

Supporting Information

Synthesis of water-soluble ionic terpolymers by inverse microemulsion and solution polymerization methods

Evelyn Y. Calvillo-Muñoz[a], Araceli Vega-Paz[b], Diego Guzman-Lucero[b], Irina V. Lijanova[a], O. Olivares-Xometl[c], Natalya V. Likhanova[b*]

[a]Instituto Politécnico Nacional, CIITEC, Cerrada Cecati S/N, Colonia Santa Catarina de Azcapotzalco, CP 02250, CDMX, México.

[b]Instituto Mexicano del Petróleo, Eje Central Norte Lázaro Cárdenas No. 152, col. San Bartolo Atepehuacan, G. A. Madero, 07730 CDMX, México.

[c]Benemérita Universidad Autónoma de Puebla, Facultad de Ingeniería Química, Av. San Claudio y 18 Sur, Ciudad Universitaria. Col. San Manuel, 72570 Puebla, Puebla, México.

* e-mail: nvictoro@imp.mx

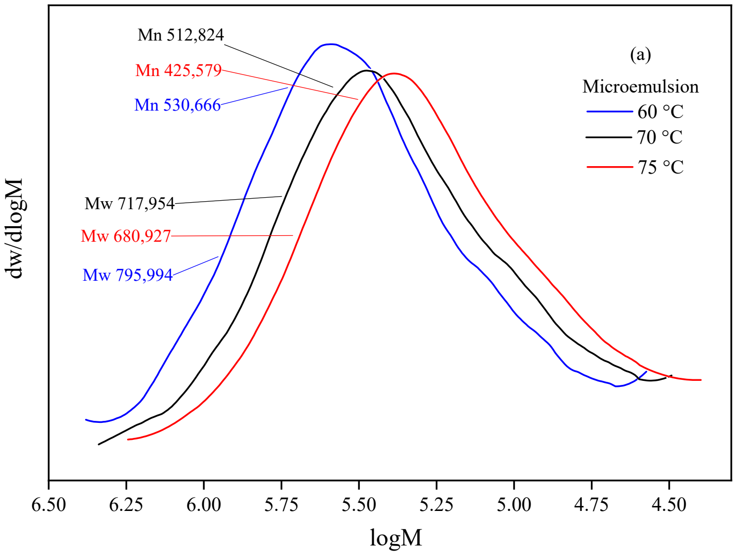

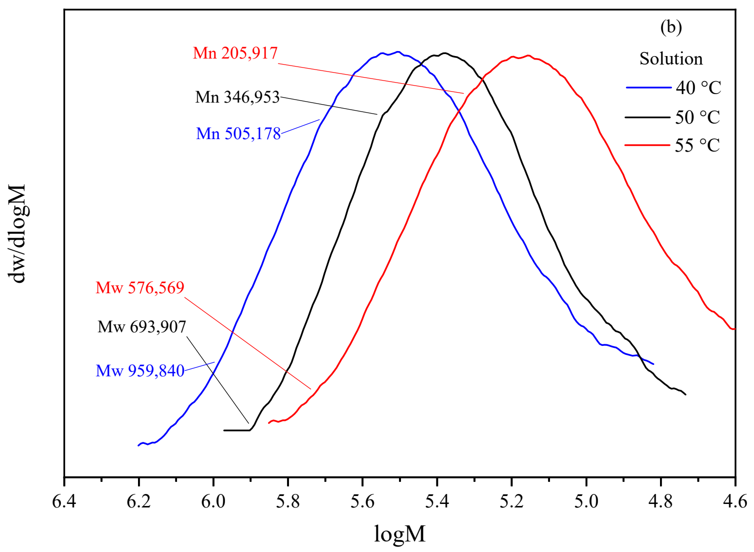

Figure S1. GPC of terpolymers by a) microemulsion method, b) solution method.
